# Sex-specific variation in R-loop formation in *Drosophila melanogaster*

**DOI:** 10.1371/journal.pgen.1010268

**Published:** 2022-06-10

**Authors:** Timothy J. Stanek, Weihuan Cao, Rohan M Mehra, Christopher E. Ellison

**Affiliations:** 1 Department of Genetics, Human Genetics Institute of New Jersey, Rutgers, The State University of New Jersey, Piscataway, New Jersey, United States of America; 2 Department of Pathology, Robert Wood Johnson Medical School, Piscataway, New Jersey, United States of America; UNITED STATES

## Abstract

R-loops are three-stranded nucleotide structures consisting of a DNA:RNA hybrid and a displaced ssDNA non-template strand. Previous work suggests that R-loop formation is primarily determined by the thermodynamics of DNA:RNA binding, which are governed by base composition (e.g., GC skew) and transcription-induced DNA superhelicity. However, R-loops have been described at genomic locations that lack these properties, suggesting that they may serve other context-specific roles. To better understand the genetic determinants of R-loop formation, we have characterized the *Drosophila melanogaster* R-loop landscape across strains and between sexes using DNA:RNA immunoprecipitation followed by high-throughput sequencing (DRIP-seq). We find that R-loops are associated with sequence motifs that are G-rich or exhibit G/C skew, as well as highly expressed genes, tRNAs, and small nuclear RNAs, consistent with a role for DNA sequence and torsion in R-loop specification. However, we also find motifs associated with R-loops that are A/T-rich and lack G/C skew as well as a subset of R-loops that are enriched in polycomb-repressed chromatin. Differential enrichment analysis reveals a small number of sex-biased R-loops: while non-differentially enriched and male-enriched R-loops form at similar genetic features and chromatin states and contain similar sequence motifs, female-enriched R-loops form at unique genetic features, chromatin states, and sequence motifs and are associated with genes that show ovary-biased expression. Male-enriched R-loops are most abundant on the dosage-compensated X chromosome, where R-loops appear stronger compared to autosomal R-loops. R-loop-containing genes on the X chromosome are dosage-compensated yet show lower MOF binding and reduced H4K16ac compared to R-loop-absent genes, suggesting that H4K16ac or MOF may attenuate R-loop formation. Collectively, these results suggest that R-loop formation in vivo is not fully explained by DNA sequence and topology and raise the possibility that a distinct subset of these hybrid structures plays an important role in the establishment and maintenance of epigenetic differences between sexes.

## Author summary

R-loops are DNA:RNA hybrid structures that act as important regulators of gene expression and genomic stability and whose dysregulation can contribute to diseases such as neurological disorders and cancer. Here, we utilize DNA:RNA immunoprecipitation followed by high-throughput sequencing (DRIP-seq) to assess the sex-specific variability in R-loop formation in *Drosophila melanogaster* adults. Most R-loops are found at simple repeats in regions of high transcriptional activity such as active chromatin states, 5’UTRs, tRNAs, and topologically associating domain boundaries. In both sexes, we find that R-loops are more common on the X chromosome compared to autosomes, likely due to an increased density of X-linked simple repeats that favor R-loop formation. While R-loops are largely conserved between sexes, we uncover a small but significant subset of sex-biased R-loops. Female-enriched R-loops are associated with genes showing ovary-biased expression and form at unique genome features, compared to other R-loops. Male-enriched R-loops occur preferentially at dosage-compensated genes on the X chromosome, yet surprisingly, show reduced levels of the marker of dosage compensation, H4K16ac, raising the possibility that the H4K16ac histone modification may attenuate R-loop formation. Our identification of sex-biased R-loops suggests a specialized role for these structures in establishing and maintaining sex-specific epigenetic programs.

## Introduction

Within the nucleus, the mechanical processes driving transcription must strike a balance between providing the cell sufficient transcripts for survival and the inherent danger to genome stability via induction of torsional stress. One mechanism by which cells regulate transcription and relieve said stress is the formation of R-loops. R-loops form when RNA invades double-stranded DNA and binds the template strand, creating a DNA:RNA hybrid and displacing the non-template strand. R-loops have been associated with a variety of biological processes and are implicated in essential aspects of gene regulation as well as genome stability [1,2]. Persistent dysregulation of R-loop maintenance can result in replication stress, DNA double-strand breaks, and chromosomal rearrangements that contribute to diseases such as neurological disorders [3] and cancer [4–7].

On a mechanistic level, there is emerging evidence that R-loop formation may be primarily driven by a combination of DNA sequence and DNA topology. At the sequence level, previous work has shown that G-rich sequences and sequences exhibiting GC skew are prone to R-loop formation [8]. DNA:RNA basepairing is more energetically favorable than DNA:DNA basepairing for G-rich and G/A-rich sequences [9], whereas transcription of linear DNA molecules exhibiting GC skew or CpG islands has been shown to lead to R-loop formation in vitro [10].

In terms of DNA topology, R-loops are known to form in response to transcription and replication-induced torsional stress in double-stranded DNA [11]. R-loop formation significantly absorbs negative superhelicity upstream of advancing polymerase complexes, functioning as a complement to DNA topoisomerase I and DNA gyrase in managing torsional stress [12–15]. Subsequent resolution of R-loops releases this stored negative superhelicity, inducing local changes such as strand separation or histone binding and potentially priming gene promoters for successive rounds of Pol II binding and firing. Such releases of superhelicity have also been shown to facilitate long-range changes in chromatin architecture such as altered promoter-enhancer contacts and loop extrusion [16,17]. Together, these prior studies suggest that R-loops are most likely to form at thermodynamically favorable regions of the genome, which are largely denoted by the base composition or the torsional state of a particular locus.

A relationship between R-loops and DNA torsion is further supported by multiple studies that document a correlation between R-loop formation and high rates of transcription, which leads to negative supercoiling upstream of the translocating polymerase. For example, in yeast strains lacking RNase H activity, R-loops have been detected at Pol III-transcribed genes, such as tRNAs and small nuclear RNAs, likely due to their high expression levels [18]. Furthermore, R-loops were found to be enriched at genes proximal to topologically associating domain (TAD) boundaries, which are known to be highly transcribed [19]. However, rather than being passive byproducts of transcription, there is evidence that R-loops are involved in specific mechanisms of gene regulation. R-loop formation may aid in Pol II pausing at transcriptional start sites [20] and promote transcriptional termination by stalling the Pol II complex and mediating access of exonucleases for 3’ cleavage of polyA sites [21–23]. In murine embryonic stem cells and Drosophila embryos, R-loops have been shown to play a role in Polycomb repressive complex 1 (PRC1) and Polycomb repressive complex 2-mediated repression of Polycomb group (PcG) target genes [24,25]. R-loops have additionally been shown to form *in trans* at circular RNAs (circRNAs) to regulate splicing factor recruitment [26] and DNA repair [27], and their formation at DNA double-strand breaks and short telomere repeats regulates Rad51-mediated homology-dependent repair [28,29]. These prior findings suggest that R-loops regulate a variety of nuclear processes, and their formation is both versatile and context-specific.

In this study, we investigate the determinants of R-loop formation in Drosophila using DNA:RNA Immunoprecipitation followed by high-throughput sequencing (DRIP-seq). We characterize genome features and sequence motifs that are associated with R-loops, and we compare the location and strength of R-loops between males and females to address whether transcriptional levels are major determinants of R-loop formation. We also assess whether hypertranscription of dosage-compensated genes is associated with increased R-loop formation in males. Overall, we find a consistent positive association between gene expression level and R-loop formation within both sexes. Furthermore, for female-enriched R-loops, we find a significant association between sex-biased gene expression and sex-biased R-loop formation. By contrast, we find that male-biased R-loops are not associated with male-biased gene expression, but instead are enriched on the X chromosome and are associated with dosage-compensated genes. While male-biased and non-biased R-loops are associated with similar sequence motifs, genome features, and chromatin states, female-biased R-loops inhabit unique regions of the genome. These results suggest that, while high transcription levels may play a role in R-loop formation, they are not sufficient to determine the R-loop landscape in Drosophila. Other genetic and epigenetic factors must also be involved, lending support to the multifaceted and context-specific nature of these genomic features.

## Results

### DRIP-sequencing

To determine the characteristics that contribute to natural variation in R-loop formation, we performed DRIP-seq separately for adult males and females in two strains of *D*. *melanogaster* from the Drosophila Genetic Reference Panel (DGRP): DGRP379 and DGRP732. Standard DRIP-seq protocols employ a restriction enzyme cocktail to fragment chromatin. However, previous studies have shown that this approach biases R-loop enrichment for specific euchromatic regions of the genome [30]. To correct for this bias, we adopted a modified protocol using a specialized sonication-based fragmentation procedure that has been shown to both preserve and isolate R-loops within genomic sequences insensitive to enzymatic digestion [31] (see Methods).

**Fig 1:**
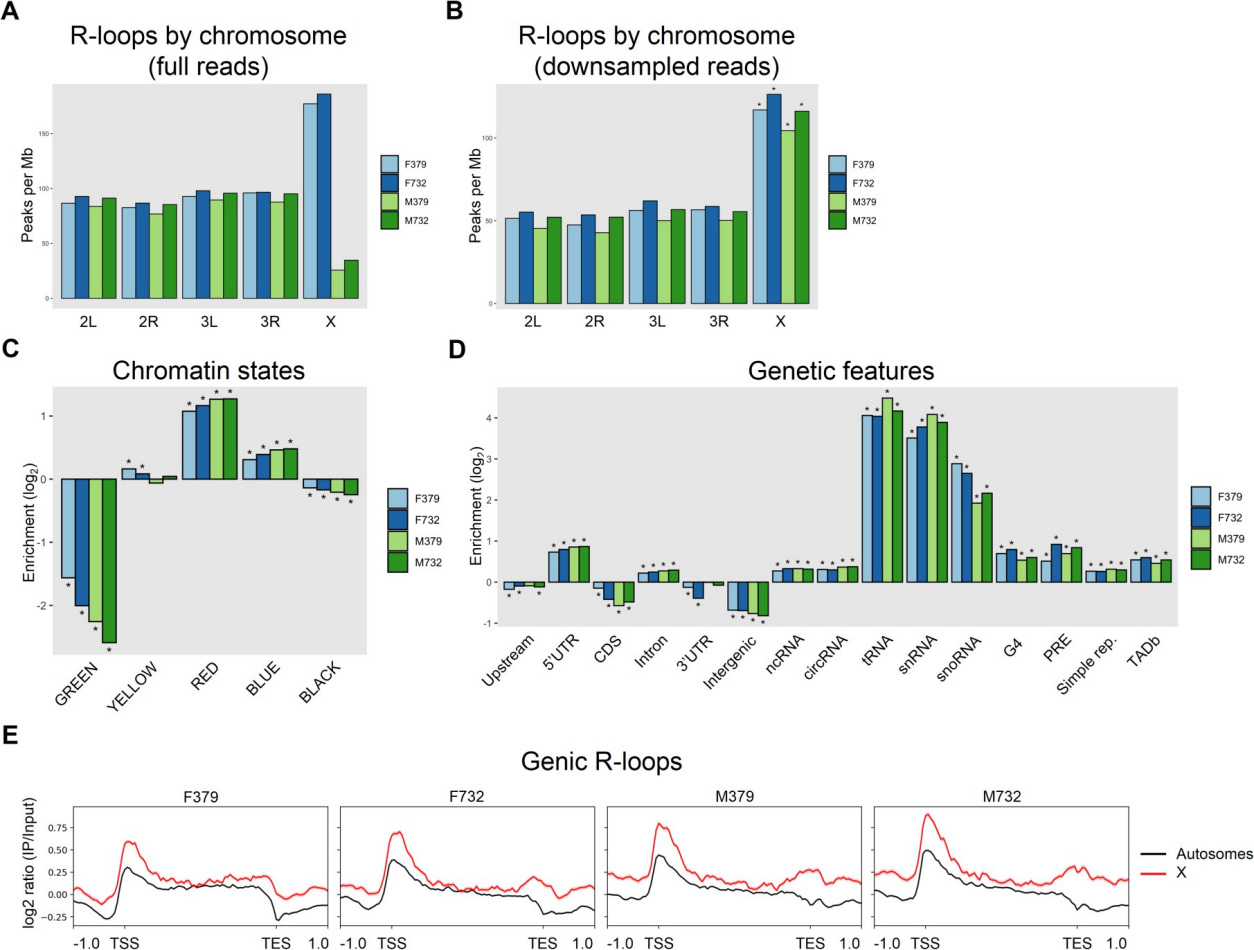
R-loop identification & feature enrichment in *D*. *melanogaster* adults. Whole adult flies from strains DGRP379 and DGRP732 were separated by sex and subjected to DRIP-sequencing to detect R-loops. (A) DRIP-seq peak density across chromosomes for the full male and female datasets (i.e. without downsampling) shows an apparent depletion of X-linked R-loops in males. (B) Downsampling all female DRIP-seq reads and autosomal male DRIP-seq reads so that all chromosome arms have similar sequencing depths shows that X-linked R-loops are enriched in both males and females (Binomial test, * = p < 2.2e-16). (C) R-loop formation at chromatin states as described in [34]. (D) R-loop formation at various genetic features. (E) Metaprofiles of R-loop signal across protein-coding genes, autosomes versus X chromosome. For Panels (C) and (D): R-loop enrichment is shown as the observed number of DRIP-seq peaks overlapping each feature (or chromatin state) divided by the expected number of peaks (see Methods). P-values were calculated via a Permutation Test with Benjamini-Hochberg correction for multiple comparisons, * = corrected p < 0.05. For Panel (E): the solid lines represent the mean DRIP-seq signal within each metagene bin, and the shading represents the standard error of the mean.

Paired-end DRIP-seq reads were aligned to primary autosomes and the X chromosome (for sequencing library information, quality control, and alignment statistics, see S1 Table). The dot chromosome was excluded from our analysis due to its high repeat density, heterochromatin content, and poor mappability [32]. After DRIP-seq peak calling, we compared peak density among chromosome arms and found a striking depletion of X-linked R-loops in males and enrichment of X-linked R-loops in females (Fig 1A). Previous work has shown that the number of identifiable ChIP-seq peaks scales with sequencing depth, without a clear saturation in many cases [33]. It is therefore possible that the depletion of DRIP-seq peaks on the male X chromosome is due to reduced sequencing depth compared to the male autosomes (and all female chromosomes), despite the fact that DRIP signal was quantified relative to an input control. In order to test this prediction, we downsampled both the male and female datasets so that all chromosomes had similar sequencing coverage (see Methods, S1 Table). The downsampled data resulted in a smaller number of peaks identified overall (as expected due to reduced sequencing depth from downsampling). However, more than twice as many peaks were identified on the male X chromosome, suggesting that the depletion of male X-linked R-loops observed in the full dataset is an artifact of lower sequencing coverage (Fig 1B). We therefore used the downsampled data for all further analyses, which resulted in 7645 to 9269 high-confidence, reproducible DRIP-seq peaks for each sample (see Methods, Fig 1B, and S2 Table).

### R-loop locations and feature enrichment

We next analyzed our high-confidence R-loop peaks for enrichment at various chromatin states [34] (Fig 1C) and genomic features (Fig 1D). R-loops are enriched primarily within the transcriptionally active RED chromatin state and are depleted in GREEN HP1-associated heterochromatin and the gene-poor BLACK repressive state. R-loops are also enriched in BLUE Polycomb-associated heterochromatin (Fig 1C), in agreement with previous studies reporting the role of R-loops in PRC-mediated repression [24,25]. Also consistent with previous studies, R-loops are enriched at 5’UTRs and introns and show no enrichment in upstream and intergenic regions (Fig 1D), highlighting their known role in Pol II-mediated transcription [8,20,35]. Surprisingly, R-loops are not enriched at 3’UTRs, in contrast with the reported association of R-loops with transcriptional termination [21,23]. R-loops are also enriched at various classes of noncoding RNAs, including circRNAs, small nuclear and small nucleolar RNAs, and tRNAs, likely due to high levels of transcription at these loci [18]. Similarly, we find that R-loops are enriched at G-quadruplexes (G4), which have been associated with open chromatin and high transcription [36], as well as TAD boundaries, where increased gene expression has been observed relative to genes within TADs [19]. Finally, we found that R-loops are enriched at simple repeats (sr), consistent with previous findings [37]. These patterns of R-loop enrichment and depletion are consistent between autosomes and the X chromosome (S1A Fig), supporting the broad role of R-loop formation in active transcription in males and females.

Metagene analysis of gene-associated R-loops reveals that at autosomal genes, R-loops appear most abundant just downstream of the transcriptional start site (TSS) and are absent from the 3’UTR, while X chromosome-associated R-loops show increased signal both across the entire gene region and at the 3’UTR (Figs 1E and S1B). These distinct profiles are observed in both sexes, suggesting that increased R-loop signal on the X chromosome occurs independently of dosage compensation-associated hypertranscription. Examination of R-loop peaks by chromosome shows that, while autosomal R-loops are present at similar levels across all samples, there is a significant enrichment of R-loops on the X chromosomes in both sexes (Binomial test P < 2.2e-16)(Fig 1B). These data indicate that R-loops tend to be associated with specific genome features and chromatin states, and their location is largely conserved between individual strains and across sexes, with the X chromosome consistently showing higher R-loop DRIP signal and peak density relative to autosomes.

### Differential enrichment and motif analysis

**Fig 2:**
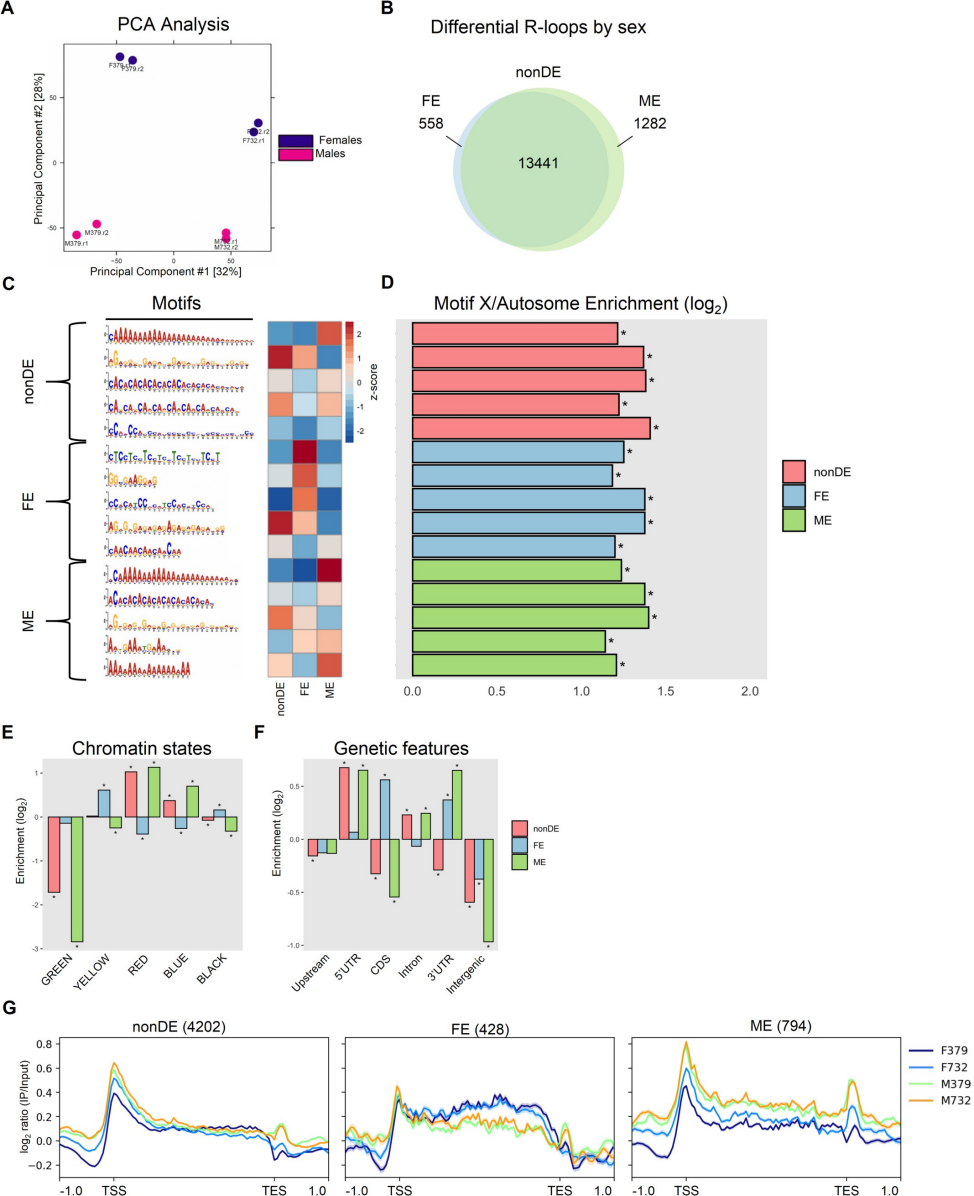
Differential enrichment and motif analysis of R-loops. (A) PCA analysis of DRIP conditions. (B) Venn diagram of non-differentially enriched (nonDE) and sex-biased (Female Enriched [FE] and Male Enriched [ME]) R-loops as identified by DiffBind. (C) STREME motif analysis by DE group; the top 5 motifs from each DE group are represented graphically (left), with z-score enrichment for each motif across DE groups plotted in the heatmap (right). (D) Motif enrichment on the X chromosome versus autosomes, plotted as log2 motifs per Mb. Binomial test, * = p < 0.001. (E) R-loop formation at chromatin states as described in [34]. (F) R-loop formation at various genetic features. (G) Metaprofiles of R-loop signal at genes within each DE group. For Panels (E) and (F): R-loop enrichment is shown as the observed number of DRIP-seq peaks overlapping each feature (or chromatin state) divided by the expected number of peaks (see Methods). P-values were calculated via a Permutation Test with Benjamini-Hochberg correction for multiple comparisons, * = corrected p < 0.05. For Panel (G): the solid lines represent the mean DRIP-seq signal within each metagene bin, and the shading represents the standard error of the mean.

Despite fewer overall R-loop peaks identified in males, gene-associated R-loop signal is higher in males compared to females on both autosomes and the X chromosome (Figs 1E and S1B). To assess sex-specific variation in R-loop formation, we subjected R-loop peaks to differential enrichment analysis using Diffbind [38]. Principal component analysis (PCA) of Diffbind peaks shows that 60% of the variation in R-loop location is captured by PC1 and PC2, which are associated with strain and sex, respectively (Fig 2A). Comparing R-loop profiles by sex via DiffBind, we identified 13,441 shared R-loops (non-differentially enriched, nonDE), 558 R-loops enriched in females (female-enriched, FE) and 1,282 R-loops enriched in males (male-enriched, ME) (Fig 2B). Overall, ~12% of R-loops are sex-biased, with more than twice as many male-biased R-loops compared to female-biased R-loops.

Motif analysis across these differentially enriched R-loop-containing loci reveals several interesting aspects of sex-biased R-loops. First, many of the most enriched motifs across all three DE groups comprise simple repeats (Fig 2C). Although some motifs contain classical GC skew [8,21] ((CCMM)_n_ in the nonDE group and ‘GGCGAAGGAG’ and (CTC)_n_ in the FE group), several enriched motifs lack such skew and instead display no skew ((CACA)_n_ in the nonDE and ME groups and (AGAG)_n_ in the FE group) or AT-skew (poly-A tracts in the nonDE and ME groups) (Fig 2C). R-loop formation at poly-A tracts specifically has been linked to high gene expression [31]. These variations in R-loop sequence favorability have been observed between model organisms, where R-loops preferentially form at GC-rich sequences in mammals, at AT-rich sequences in *Arabidopsis*, and at both sequence classes in yeast [8,21,39,40]. Interestingly, the nonDE and ME groups share three of their five top enriched motifs, whereas only one motif is shared between the nonDE and FE groups (Fig 2C). All motifs identified are significantly enriched on the X chromosome compared to autosomes (Fig 2D), consistent with our observed enrichment of R-loops on the X chromosome (Fig 1B).

The unique motifs found at FE R-loops suggest that they may occupy genomic features distinct from the nonDE and ME R-loops. Assigning these sex-biased R-loops to known chromatin states [34] reveals similar enrichments between nonDE and ME R-loops (Fig 2E). By contrast, FE R-loops show the opposite pattern of enrichment or depletion in nearly every chromatin state, with enrichment primarily in the YELLOW active state and the BLACK repressive state (Fig 2E). Genetic feature analysis similarly shows that FE R-loops preferentially form at loci distinct from the nonDE and ME groups, most notably within the CDS of genes (Fig 2F). Metagene analysis of these differentially enriched R-loops at genic loci further support the uniqueness of the FE group: the DRIP signal at FE genes is depleted from the TSS and concentrated across the CDS, opposite from that seen for the nonDE and ME groups (Figs 2G and S2A). Furthermore, gene ontology analysis reveals that ME R-loops form at genes associated with developmental and regulatory processes, whereas FE R-loops form at genes associated with translation and biosynthesis, including a large number of ribosomal proteins (S3 Table).

Given the relationship between high rates of transcription and R-loop formation, we next sought to determine whether the ME and FE R-loops occur at genes that show sex-biased expression patterns. We performed RNA-seq from whole flies for the same four samples used for DRIP-seq and found that, surprisingly, ME and FE R-loop-containing genes show similar expression patterns in both sexes (S2B Fig). To address the possibility that the ME and FE genes are differentially expressed in specific tissues, we used the FlyAtlas database to investigate their expression patterns in ovaries, testes, and brain, tissues where sex-biased gene expression has previously been characterized (S2C Fig) [41–43]. In contrast to our whole fly expression data, genes containing female-enriched R-loops are expressed at significantly higher levels in ovaries compared to genes with male-enriched R-loops and conserved R-loops (S2C Fig, left panel). Conversely, genes containing male-enriched R-loops are expressed at significantly lower levels in the testes compared to genes with female-enriched R-loops and conserved R-loops (S2C Fig, center panel). In the brain, genes from all three categories (FE, ME, and nonDE) are expressed at similar levels (S2C Fig, right panel). Taken together, these observations suggest that sex-specific utilization of R-loops in Drosophila is only partly explained by sex-biased gene expression.

Because the gonads make up a large percentage of the adult body, we reasoned that the DRIP signal from whole adult flies is likely similar to that from gonads alone. To test this prediction, we performed DRIP-seq in ovaries dissected from adult females and found strong, highly significant correlations between the female whole fly samples and ovary samples for both nonDE and FE peaksets from the adult data (Spearman’s rho > = 0.72 and P < 2.2e-16 in all comparisons, S2D Fig).

### X chromosome-specific R-loop enrichment

**Fig 3:**
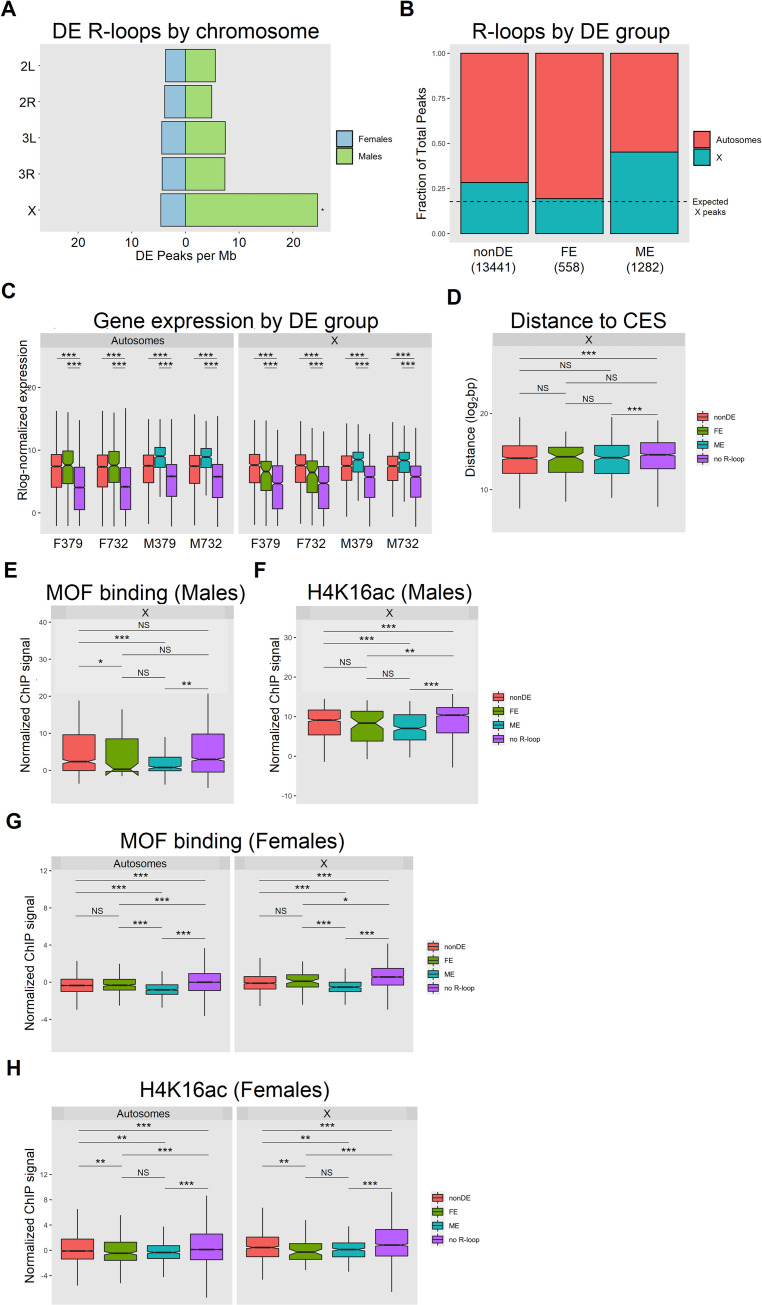
X chromosome-specific R-loop enrichment. (A) Differentially enriched R-loops by chromosome, plotted as R-loop peaks per Mb. Binomial test, * = p < 0.001. (B) Differentially enriched R-loop frequency on autosomes versus X chromosome, plotted as a fraction of total R-loops per DE group. (C) Gene expression analysis of R-loop-containing genes by DE group versus R-loop-absent (no R-loop) genes, on autosomes and the X chromosome, plotted as rlog-normalized expression. Wilcoxon test, *, **, *** = p < 0.05, 0.01, 0.001. (D) Distance to chromosomal entry site (CES) on the X chromosome across DE groups, plotted in log_2_ base pairs (bp). Wilcoxon test, *** = p < 0.001. (E) MOF binding and (F) H4K16ac enrichment on the X chromosome in third-instar larva male salivary glands across DE groups. Wilcoxon test, **, *** = p < 0.01, 0.001. (G) MOF binding and (H) H4K16ac enrichment on autosomes and the X chromosome in third-instar larva female salivary glands across DE groups. Wilcoxon test, *,**,*** = p < 0.05, 0.01, 0.001.

Given the presence of sex-specific R-loop enrichment in Drosophila adults, we explored the possibility that these differences were associated with the sex chromosome and dosage compensation. As noted above, R-loops form with increased frequency on the X chromosome compared to autosomes in both males and females (Fig 1B). However, when focusing only on differentially enriched R-loops, those found on the X chromosome are enriched in males significantly more than the general X enrichment seen across all R-loops (Fig 3A and 3B). By contrast, the DE R-loops in females show no X-enrichment (Fig 3B). Given the established relationship between R-loops and transcription, this observation suggests an association of R-loops with the dosage compensation mechanism of the male X chromosome [44,45].

R-loop-containing genes are expressed at higher levels than genes with no detectable R-loops on both autosomes and the X chromosome (Fig 3C), consistent with transcription-induced R-loop formation [18,31]. On the X chromosome, this association is further supported by the significantly closer proximity of nonDE and ME R-loops to chromosomal entry sites (CES) (Fig 3D), which help initiate and propagate histone acetylation associated with the dosage compensation complex (DCC). However, analysis of a publicly available dataset of DCC machinery and its cognate modifications [46,47] shows that, in male salivary glands, both the histone acetyltransferase males-absent on the first protein (MOF) and the histone mark H4K16ac are depleted at ME genes relative to both nonDE genes and no R-loop genes (Fig 3E and 3F), despite showing evidence of dosage compensation based on gene expression (S3A Fig). These results are consistent with a positive association between transcription and R-loop formation that is possibly attenuated by high levels of the H4K16ac histone modification.

To further explore the relationship between R-loop formation and MOF/H4K16ac levels, we extended our comparison above to females. In females, the MSL complex is absent due to translational repression of *msl-2* by Sex-lethal (Sxl) [48]. Instead, the MOF-containing NSL complex acts to deposit H4K16ac at actively transcribed genes [49]. We therefore used the female R-loop data to determine whether a negative relationship exists between the presence of R-loops and MOF and H4K16ac levels, similar to what we observed on the male X chromosome. All R-loop classes (i.e., non-DE, FE, and ME) show significantly lower enrichment of MOF and H4K16ac compared to expressed genes with no R-loops, on both the autosomes and X chromosome (Fig 3G and 3H), providing additional support that R-loops are less likely to form at genomic regions with high levels of MOF/H4K16ac.

## Discussion

Our assessment of natural R-loop variation between sexes has revealed multiple insights. First, R-loops are largely associated with transcriptionally active loci: they are found in chromatin states with either broad or specific transcriptional programs. More specifically, R-loops form proximal to the TSS, at multiple classes of ncRNAs and G-quadruplexes, and at TAD boundaries where transcriptionally active loci have been shown to reside [19]. Mechanistically, this enrichment is supported by the role R-loops play in relieving transcription-mediated torsional stress [11,14,16]. To our surprise, we failed to observe R-loop enrichment at 3’UTRs in Drosophila, despite previous studies implicating R-loop formation in transcriptional termination [22,23]. At some of these features, such as circRNAs, R-loop formation can occur *in trans* [26,27], yet whether the circRNA-associated R-loops detected in this study also form *in trans* remains to be determined. In addition to an association between R-loops and overall gene transcription, our observation of R-loop enrichment within the BLUE Polycomb-regulated chromatin state and at PREs in Drosophila supports previously established roles that R-loops play in Polycomb-mediated gene repression [24,25,50].

Our differential enrichment analysis confirms the strong conservation of the R-loop landscape between individuals and sexes, in line with previous comparisons across human and murine cell lines [30]. Additionally, the majority of R-loop-associated DNA motifs that we identify are simple repeats. The Drosophila X chromosome has previously been shown to be enriched for simple repeats, in general, compared to autosomes [51], suggesting that the sequence content of this chromosome may explain, at least in part, our observation of increased R-loop formation on the X. However, we note that R-loop density is roughly 2-fold higher on the X compared to the autosomes whereas the R-loop motifs all show less than 1.5-fold enrichment on the X, suggesting that other factors may also be involved.

Despite the overall conservation of the R-loop landscape between sexes, we also observe a subset of sex-biased R-loops, suggesting some level of specialization for their formation and function. The enrichment of nearly half of all male-enriched R-loops on the male X chromosome likely reflects its hypertranscribed, dosage-compensated state. Furthermore, the relationship between transcription and R-loop formation is maintained even within the X chromosome: R-loop-containing genes on the male X exhibit higher gene expression levels than X-linked genes lacking R-loops. It seems paradoxical, then, that canonical markers of active transcription deposited by the MSL and NSL complexes are reduced at R-loop-containing genes compared to genes with no R-loops. One possible explanation is that H4K16 acetylation may subtly disfavor R-loop formation. Indeed, previous studies have established a role for the DCC and H4K16ac in reducing negative superhelicity and disordering dosage-compensated chromatin to encourage DNA binding protein activity and Pol II loading [52,53]. This reduction in negative superhelicity could make R-loop formation less energetically favorable at highly acetylated genes or genes strongly bound by the DCC. However, such a relationship does not mean these two modifications should be mutually exclusive. Instead, the propensity for either R-loops or histone acetylation to relieve transcription-associated superhelicity is likely affected by multiple aspects of the local chromatin environment.

For the genes associated with female-enriched R-loops, their ovary-biased expression raises the possibility that the FE R-loops form specifically in ovaries, yet the function of these R-loops remains elusive. Their unique motifs and association with ribosomal protein and translation-related genes distinguish them from the nonDE and ME groups. The sequence-specific transcription factor binding protein (M1BP) regulates transcription of ribosomal protein genes [54] and is maternally deposited and highly expressed in early embryos [55], but whether this contributes directly to the enrichment of FE R-loops at ribosomal and translation-associated genes remains to be seen. More locally, at genic loci, the distribution of FE R-loops across the gene body diverges from the typical TSS enrichment observed in the other DE groups. Additional scrutiny of these intragenic R-loops is required to determine their function in comparison with the more typical promoter and terminator-associated R-loops. Previous studies have demonstrated a role for R-loops regulating histone modifications and chromatin remodeling complex binding [56–58], raising the possibility that these female-enriched R-loops, rather than forming in response to DNA superhelicity, instead serve a distinct and context-specific regulatory function.

In summary, this work provides insight into the genome features, sequence motifs, and natural variation of the R-loop landscape in Drosophila. Our results are consistent with transcription rate, DNA torsion, and base composition being important determinants of R-loop formation. However, none of these properties fully explains the sex-biased R-loops that we identify, suggesting that other genetic or epigenetic mechanisms are involved in their formation. Further study of these male and female-biased R-loops will provide insight into their role in the establishment and maintenance of epigenetic differences between sexes.

## Materials and methods

### S1-DRIP-seq

As R-loops are known to be sensitive to sonication-induced degradation [30,31], we digested purified chromatin with S1 nuclease to remove the non-template strand prior to sonication, which has been shown to protect R-loop integrity through the sonication process [31]. Purification and sequencing of R-loops was performed as described in [31], with modifications. Briefly, whole adult DGRP379 and DGRP732 flies were separated by sex and homogenized, and genomic DNA (gDNA) was extracted using the DNEasy Blood & Tissue Kit (Qiagen). Extracted gDNA was digested with S1 nuclease to remove the non-template strand of DNA:RNA hybrids and sonicated with a Covaris S2 (Covaris) to an average fragment size of 100–300 bp. Consistent fragment size distribution across samples was confirmed via capillary electrophoresis (Agilent Fragment Analyzer, S4 Table). R-loops were immunoprecipitated with the S9.6 antibody (EMD Millipore) conjugated to Dynabeads Protein A (ThermoFisher), eluted with 1% SDS, and purified with the ChIP DNA Clean & Concentrator kit (Zymo Research). Illumina libraries from IP and Input samples were prepared with the DNA SMARTer ThruPLEX DNA-Seq kit (Takara Bio) and SMARTer DNA Unique Dual Index kit (Takara Bio).

For DRIP-seq of ovaries, ovaries were dissected from 2 to 10-days-old w1118 females (90 females per replicate) and homogenized in cold PBS. Genomic DNA was extracted and processed for S1-DRIP-seq as described above.

### R-loops alignment and peak-calling

Reads were trimmed with trimmomatic [59] with the following options: “PE -phred33 ILLUMINACLIP:TruSeq3-PE.fa:2:30:10:8:TRUE LEADING:15 TRAILING:15 SLIDINGWINDOW:3:10 MINLEN:36”, and aligned to the *D*. *melanogaster* reference genome assembly FlyBase version 6 [60,61] using bowtie2 [62] with the following options: “--no-mixed --no-discordant --dovetail --phred33 -X 1000”.

The ENCODE group has found that there is a consistent relationship between the number of ChIP-seq peaks identified and sequencing depth, without a clear saturation in most cases, due to an increased ability to identify low-affinity sites with increased sequencing depth [33]. Reads aligned to all female chromosomes and all male autosomes were therefore downsampled by 50% using samtools [63] with the following options: “-b -s 0.5”. High-confidence R-loop peaks were called using MACS2 [64] with the following options: “callpeak -f BAMPE -g dm -B -p 1e-3 -t IP.bam -c Input.bam”. To ensure reproducibility of R-loop loci between individual replicates of each condition, we employed the Irreproducibility Discovery Rate (IDR) framework [65] to identify a set of high-confidence DRIP peaks from all peaks called by MACS2. Pseudoreplicate and self-pseudoreplicate ratios confirmed that the shared peaks identified in each condition were reproducible (S2 Table). We assessed the chromosomal enrichment of R-loops by counting the number of peaks present on each of the 5 major chromosome arms of *D. melanogaster*, for each of our DRIP-seq samples. To control for the differences in length among the chromosome arms, we normalized the peak counts by chromosome length (in millions of basepairs, Mb). To determine whether the increased number of peaks observed on the X chromosome was statistically significant, we used a binomial test to test the null hypothesis that there is no difference in peak density between the X chromosome and autosomes.

### Features overlap

DRIP peaks from each sample were intersected with genomic features and chromatin states using bedtools intersect [66] and plotted as log2 enrichment of observed counts/expected counts, where observed counts comprised the total number of overlaps between a specific peakset and each feature or chromatin state, and expected counts comprised the average number of overlaps between each feature or chromatin state and 10000 iterations of shuffled R-loop peaks using bedtools shuffle with the “-chrom” option to preserve peak width and chromosomal location; reads aligning to tRNAs were counted only once per unique tRNA gene sequence. For each feature type, two-sided permutation test P-values were calculated as the proportion of permutations showing the same or more overlaps as the observed counts (i.e., enrichment) or the same or fewer overlaps as the observed counts (i.e., depletion). P-values were then adjusted for multiple hypothesis testing using the Benjamini-Hochberg correction. Genomic coordinates for gene, tRNA, snRNA, and snoRNA features were derived from FlyBase genome annotations [67]. Genomic coordinates for custom features were derived from the following studies: chromatin states [34], circRNAs [68], Polycomb-responsive elements [25], simple repeats previously identified from Repeatmasker [69], G-quadruplexes identified using pqsfinder (min_score = 52) [70], and strain-specific TAD boundaries identified from previously published Hi-C data [71] using HiC-Explorer [72].

### Differential enrichment analysis

Differential R-loops were identified using DiffBind [38]. Briefly, IDR-called peaks were used to create a consensus peakset (bUseSummarizeOverlaps = TRUE, summits = FALSE) composed of DRIP-seq peaks found in at least two of the four samples (i.e., F379, F732, M379, M732). Sex-biased R-loops were subsequently identified using this consensus peakset (bContrasts = TRUE, adjusted p-value < 0.1).

### Metagene analysis

Metaprofiles of expressed R-loop-containing genes were generated using deeptools [73] computeMatrix with the following options: “scale-regions --transcriptID mrna --skipZeros -p 20 -b 1000 -a 1000 --regionBodyLength 3000 –binSize 50”, followed by plotProfile with the following options: “--perGroup --plotType se”.

### Motif identification and enrichment

Motif analysis was performed using STREME (MEME suite) [74] with the following p-value thresholds across DE groups: nonDE -pvt 1e-10, FE -pvt 1e-2, ME -pvt 1e-2. For comparison of motif enrichment across enrichment groups, the top five motifs from each group were analyzed using *gimme maelstrom* from GimmeMotifs [75] using the “--no-filter” option. To assess autosome-vs-X chromosome enrichment of these identified motifs, FIMO (MEME suite) [76] was used to identify all occurrences of each DNA motif on the 5 major chromosome arms. The ratio of motif occurrences per Mb on the X chromosomes versus autosomes was used to determine motif enrichment. Statistical significance was determined via a binomial test of the null hypothesis that there is no difference in motif density between the X chromosome and autosomes.

### Adult females versus ovaries read coverage comparison

Female adult DRIP-seq and ovaries DRIP-seq read coverage at loci from nonDE and FE peaksets was compared using deeptools [73] multiBamSummary with the following options: “--genomeChunkSize 129941135 --outRawCounts”. Output of raw read counts per loci was normalized to total read count per sample and plotted as reads per megabase (Mb). Statistical significance was determined using Spearman correlation coefficient.

### RNA-seq and gene expression analysis

We used ~10 whole adult DGRP379 and DGRP732 males and females. Flies were homogenized with an electric pestle in DNA/RNA Shield solution (Zymo Research). Homogenized tissue was digested with Proteinase K and RNA was purified with the Zymo Quick-RNA Plus Kit (Zymo Research). Ribosomal RNAs were removed using siTools rRNA depletion Kit (Galen Laboratory Supplies) and MyOne Streptavidin C1 Dynabeads (ThermoFisher) (#65001). Ribosomal RNA-depleted RNA was purified using the RNA Clean and Concentrator-5 kit (Zymo Research). Illumina libraries were generated using NEBNext Ultra II Directional RNA Library Prep Kit for Illumina (NEB).

RNA sequencing reads were first aligned to the FlyBase r6.27 rRNA sequences using HISAT2 [77]. Non-ribosomal sequences were subsequently aligned to FlyBase r6.27 transcript sequences using htseq-ct [78]. Counts were filtered to include only expressed transcripts using DESeq2 [79] (rowSums(DESeqDataSetFromHTSeqCount) > = 1), which were subsequently normalized using rlog transformation (blind = TRUE) in DESeq2 [79].

### FlyAtlas microarray expression analysis

Microarray expression data were downloaded from http://flyatlas.org/. Genes were separated by differential enrichment group: “nonDE,” “FE,” “ME,” and “no R-loop,”. The minimum gene expression threshold for each tissue was determined by detectable expression in at least two of four microarrays (columns “OvaryPresent”, “TestisCall”, or “BrainPresent” ≥ 2).

## Supporting information

S1 Fig Related to Fig 1, R-loop identification & feature enrichment in *D*. *melanogaster* adults. (A) R-loop formation at various genetic features on autosomes (upper panel) and the X chromosome (lower panel). R-loop enrichment is shown as the observed number of DRIP-seq peaks overlapping each feature (or chromatin state) divided by the expected number of peaks (see Methods). P-values were calculated via a Permutation Test with Benjamini-Hochberg correction for multiple comparisons, * = corrected p < 0.05. (B) Metaprofiles of R-loop signal across protein-coding genes (from Fig 1E), overlapped by condition, grouped by chromosome. The solid lines represent the mean DRIP-seq signal within each metagene bin and the shading represents the standard error of the mean.
(TIF)

S2 Fig Related to Fig 2, Differential enrichment and motif analysis of R-loops. (A) Metaprofiles of R-loop signal across protein-coding genes (from Fig 2G), separated by condition and by DE group. The solid lines represent the mean DRIP-seq signal within each metagene bin, and the shading represents the standard error of the mean. (B) Gene expression analysis of R-loop-containing genes by sex, separated by DE group versus R-loop-absent (no R-loop) genes across all chromosomes, autosomes, and the X chromosome, plotted as rlog-normalized expression. (C) Microarray gene expression analysis from the FlyAtlas [41] of R-loop-containing genes across tissues, separated by DE group. Wilcoxon test, **,*** = p < 0.01, 0.001. (D) DRIP-seq normalized read coverage of whole female flies and ovaries. The black line in each plot represents a slope of 1 with intersect at 0. Spearman’s rho = 0.72, 0.76 and p < 2.2e-16, 2.2e-16 [nonDE peaks-F379, F732], Spearman’s rho = 0.74, 0.79 and p < 2.2e-16, 2.2e-16 [FE peaks-F379, F732].
(TIF)

S3 Fig Related to Fig 3, X chromosome-specific R-loop enrichment. (A) Male-to-female gene expression ratios by DE group on autosomal and X chromosome genes. Wilcoxon test, **,*** = p < 0.01, 0.001.
(TIF)

S1 Table DRIP-seq sequencing statistics and down-sampling by chromosome. (XLSX)

S2 Table IDR analysis determines the rate of reproducibility between replicates. (XLSX)

S3 Table GO Enrichment analysis of sex-biased R-loops. (XLSX)

S4 Table DRIP-seq libraries Agilent Fragment Analyzer statistics. (XLSX)
