# Supplementary figures and images for: Sex-specific variation in R-loop formation in Drosophila melanogaster
Source: PLoS Genet. 2022 Jun 10;18(6):e1010268. doi: 10.1371/journal.pgen.1010268 (PMC9223372; doi:10.1371/journal.pgen.1010268)

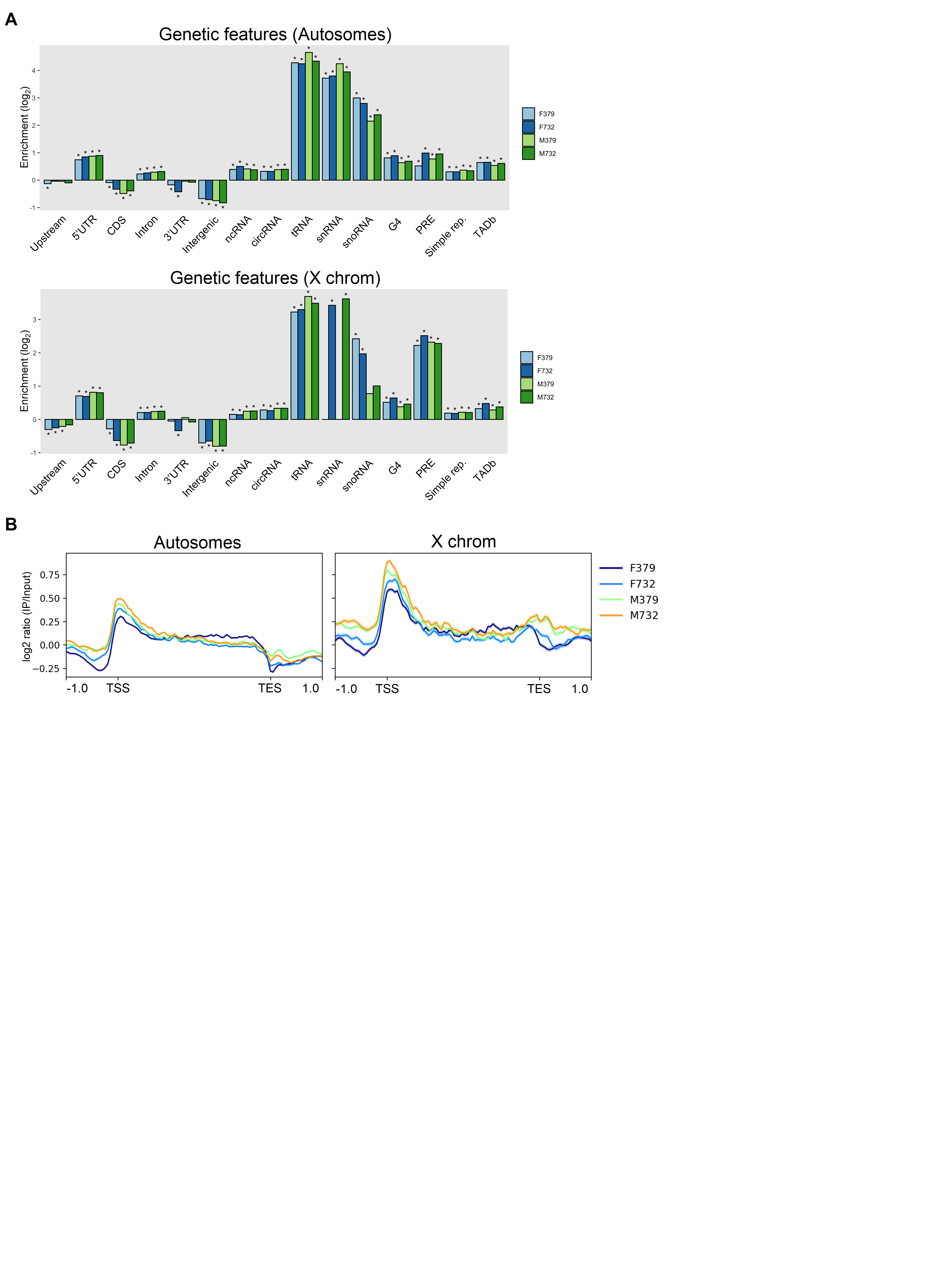

Supplement: S1 Fig — (A) R-loop formation at various genetic features on autosomes (upper panel) and the X chromosome (lower panel). R-loop enrichment is shown as the observed number of DRIP-seq peaks overlapping each feature (or chromatin state) divided by the expected number of peaks (see Methods). P-values were calculated via a Permutation Test with Benjamini-Hochberg correction for multiple comparisons, * = corrected p < 0.05. (B) Metaprofiles of R-loop signal across protein-coding genes (from Fig 1E), overlapped by condition, grouped by chromosome. The solid lines represent the mean DRIP-seq signal within each metagene bin and the shading represents the standard error of the mean. (TIF) [file pgen.1010268.s001.TIF]

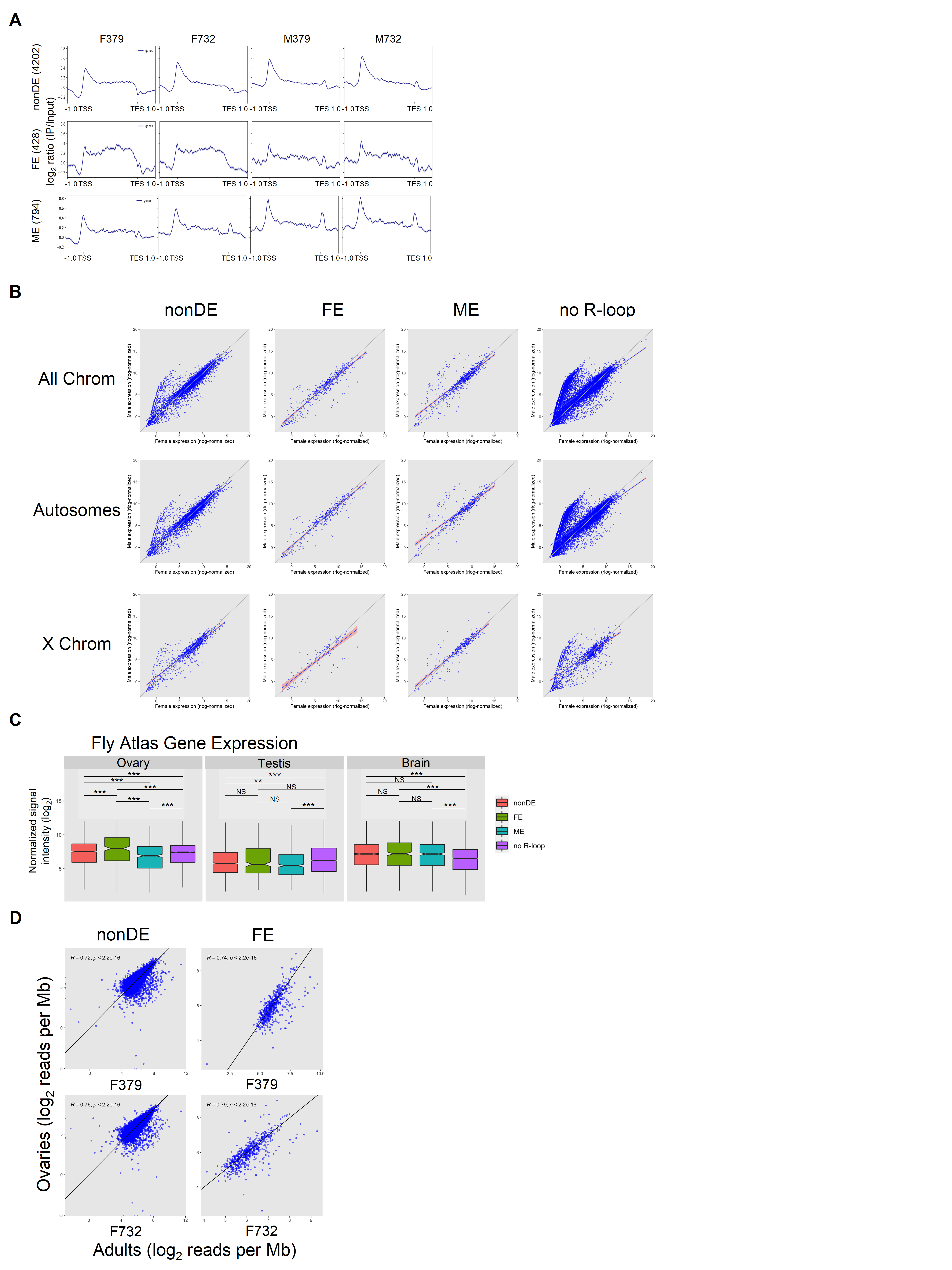

Supplement: S2 Fig — (A) Metaprofiles of R-loop signal across protein-coding genes (from Fig 2G), separated by condition and by DE group. The solid lines represent the mean DRIP-seq signal within each metagene bin, and the shading represents the standard error of the mean. (B) Gene expression analysis of R-loop-containing genes by sex, separated by DE group versus R-loop-absent (no R-loop) genes across all chromosomes, autosomes, and the X chromosome, plotted as rlog-normalized expression. (C) Microarray gene expression analysis from the FlyAtlas [41] of R-loop-containing genes across tissues, separated by DE group. Wilcoxon test, **,*** = p < 0.01, 0.001. (D) DRIP-seq normalized read coverage of whole female flies and ovaries. The black line in each plot represents a slope of 1 with intersect at 0. Spearman’s rho = 0.72, 0.76 and p < 2.2e-16, 2.2e-16 [nonDE peaks-F379, F732], Spearman’s rho = 0.74, 0.79 and p < 2.2e-16, 2.2e-16 [FE peaks-F379, F732]. (TIF) [file pgen.1010268.s002.TIF]

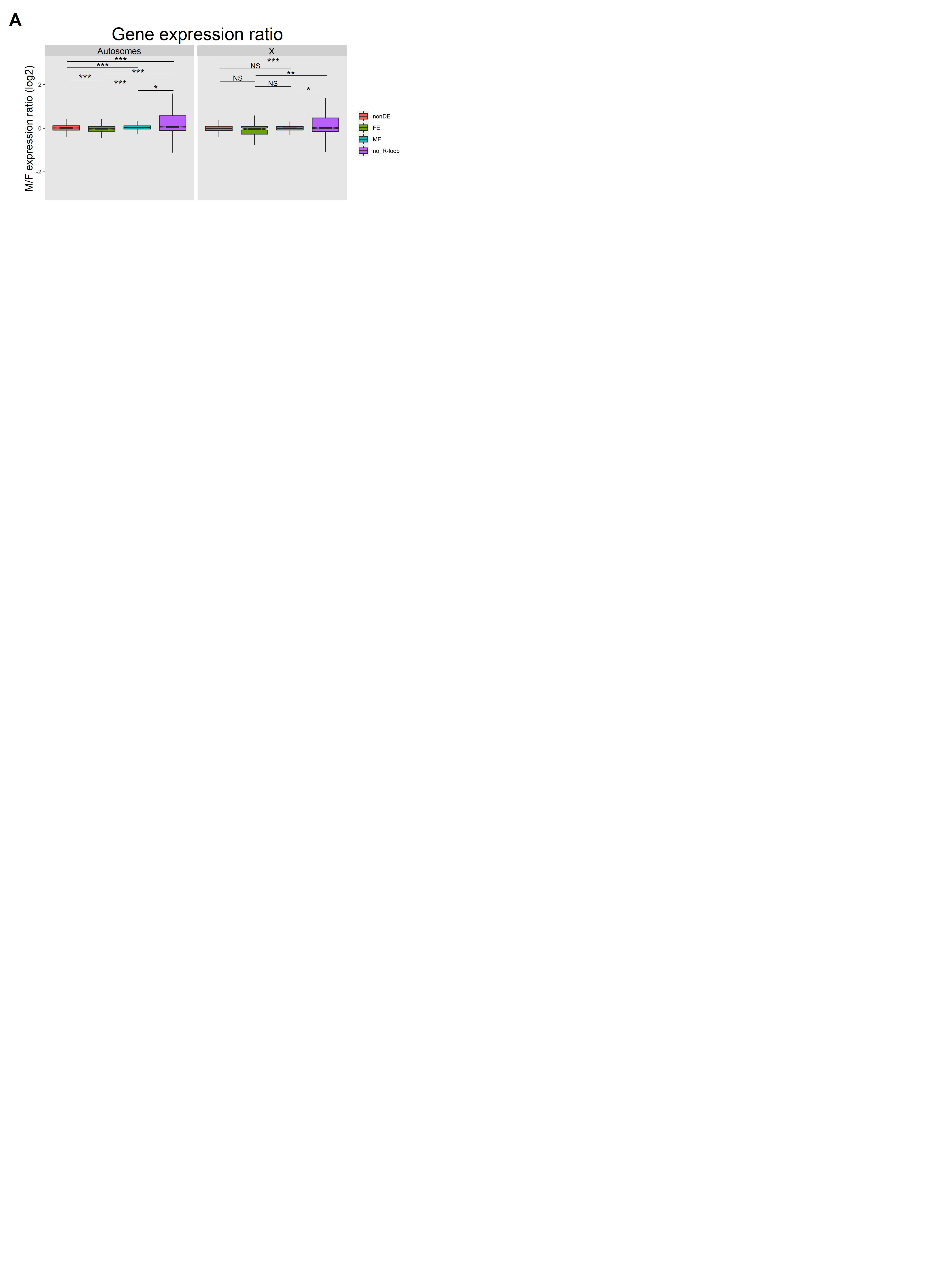

Supplement: S3 Fig — (A) Male-to-female gene expression ratios by DE group on autosomal and X chromosome genes. Wilcoxon test, **,*** = p < 0.01, 0.001. (TIF) [file pgen.1010268.s003.TIF]
